# Mesial temporal tau in amyloid-β-negative cognitively normal older persons

**DOI:** 10.1186/s13195-022-00993-x

**Published:** 2022-04-08

**Authors:** Natasha Krishnadas, Vincent Doré, Colin Groot, Fiona Lamb, Pierrick Bourgeat, Samantha C. Burnham, Kun Huang, Anita M. Y. Goh, Colin L. Masters, Victor L. Villemagne, Christopher C. Rowe, for the AIBL research group

**Affiliations:** 1 Florey Department of Neurosciences & Mental Health, The University of Melbourne, Parkville, VIC 3052 Australia; 2 Department of Molecular Imaging & Therapy, Austin Health, 145 Studley Rd, Heidelberg, VIC 3084 Australia; 3 Health and Biosecurity Flagship, The Australian eHealth Research Centre, Melbourne, Victoria Australia; 4 Health and Biosecurity Flagship, The Australian eHealth Research Centre, Brisbane, QLD Australia; 5 Department of Psychiatry, The University of Melbourne, Parkville, VIC 3010 Australia; 6 National Ageing Research Institute, Parkville, VIC 3052 Australia; 7 Florey Institute of Neuroscience & Mental Health, Parkville, VIC 3052 Australia; 8 Department of Psychiatry, University of Pittsburgh, Pittsburgh, PA USA

**Keywords:** Positron emission tomography (PET), Cognition, Aging, ^18^F-MK6240, Mesial temporal lobe, Tau

## Abstract

**Background:**

Tau deposition in the mesial temporal lobe (MTL) in the absence of amyloid-β (Aβ−) occurs with aging. The tau PET tracer ^18^F-MK6240 has low non-specific background binding so is well suited to exploration of early-stage tau deposition. The aim of this study was to investigate the associations between MTL tau, age, hippocampal volume (HV), cognition, and neocortical tau in Aβ− cognitively unimpaired (CU) individuals.

**Methods:**

One hundred and ninety-nine Aβ− participants (Centiloid < 25) who were CU underwent ^18^F-MK6240 PET at age 75 ± 5.2 years. Tau standardized uptake value ratio (SUVR) was estimated in mesial temporal (Me), temporoparietal (Te), and rest of the neocortex (R) regions and four Me sub-regions. Tau SUVR were analyzed as continuous variables and compared between high and low MTL SUVR groups.

**Results:**

In this cohort with a stable clinical classification of CU for a mean of 5.3 years prior to and at the time of tau PET, MTL tau was visually observed in 9% of the participants and was limited to Braak stages I–II. MTL tau was correlated with age (*r* = 0.24, *p* < 0.001). Age contributed to the variance in cognitive scores but MTL tau did not. MTL tau was not greater with subjective memory complaint, nor was there a correlation between MTL tau and Aβ Centiloid value, but high tau was associated with smaller HV. Participants with MTL tau had higher tau SUVR in the neocortex but this was driven by the cerebellar reference region and was not present when using white matter normalization.

**Conclusions:**

In an Aβ− CU cohort, tau tracer binding in the mesial temporal lobe was age-related and associated with smaller hippocampi, but not with subjective or objective cognitive impairment.

**Supplementary Information:**

The online version contains supplementary material available at 10.1186/s13195-022-00993-x.

## Introduction

Mesial temporal lobe (MTL) structures and their intrinsic network connectivity are essential for memory and complex cognitive processing. Intraneuronal tau aggregates (neurofibrillary tangles [NFT] and neuropil threads) in the MTL are commonly observed in brain post-mortem studies of older persons [1–3]. While observed in younger individuals [4, 5], tau deposition tends to increase with age [1, 3]. Tau aggregates are initially observed in trans-entorhinal and entorhinal (EC) regions in conjunction with cortical amyloid-β (Aβ) in Alzheimer’s disease (AD) [1, 6], where episodic memory impairment is a common presenting symptom [7]. In AD, the presence of tau (essentially high Aβ and high tau) has been more closely linked with the development of cognitive impairment than Aβ alone [8, 9]. In the absence of significant Aβ pathology, tau NFT typically restricted to the MTL, basal forebrain, brainstem, and olfactory areas have been described as primary age-related tauopathy (PART) [10]. There remains debate as to whether PART represents a unique disease entity or whether it represents an early stage of AD [10, 11].

PART identified on post-mortem examination has been associated with antemortem cognitive impairment in a subset of individuals, characterized by impairments on tests of episodic and semantic memory [12], attention [12], processing speed [12, 13], and executive function [13]. However, when present, cognitive impairment was associated with higher tau burden (Braak stages III–IV) [12, 13].

A few observational in vivo studies have reported an Aβ-independent association of entorhinal tau and worse episodic memory in older individuals who were still considered cognitively normal [14–17]. There is a shift in AD clinical trials toward the pre-clinical stages of disease, with trials aiming to screen healthy older individuals with Aβ and tau biomarkers [18, 19]. Thus, broad screening may invariably identify MTL tau in otherwise healthy Aβ-negative (Aβ−) older persons. The tau PET tracer ^18^F-MK6240 has characteristics that favor its ability to detect small quantities of MTL tau [20]. Therefore, we aimed to study the associations between MTL tau (as measured by ^18^F-MK6240 tau PET), age, hippocampal volume, cognition, and neocortical tau in Aβ− cognitively unimpaired (CU) individuals. It was hypothesized that high entorhinal/MTL tau in the absence of Aβ would be associated with age and associated with worse cognitive scores.

## Methods

### Participants

Participants from the Australian Imaging Biomarker and Lifestyle (AIBL) study of aging who completed Aβ and tau (^18^F-MK6240) PET scans before August 2021 were included in this study if they met the following criteria: (1) ≥60 years of age; (2) Aβ negative (defined as Centiloid < 25); (3) were fluent in English; (4) had completed at least 7 years of education; (5) did not have any history of neurological or psychiatric disorders, drug or alcohol abuse or dependence, or any other unstable medical condition; and (6) were deemed to be cognitively unimpaired (CU), based on their performance on a battery of cognitive assessments that AIBL participants undergo every 12 to 18 months. A multi-disciplinary clinical review panel determines whether an individual is CU, based on the available clinical and neuropsychological information. The full methodology for cohort recruitment and assessment has been described previously [21]. All relevant institutional review boards have approved this study, and written informed consent was obtained from all participants.

### Image acquisition

Tau PET imaging involved the intravenous administration of 185MBq (± 10%) of ^18^F-MK6240 with a 20-min acquisition commencing 90-min post-injection. Aβ PET imaging involved the intravenous administration of 200MBq (±10%) of ^18^F-NAV4694 with a 20-min acquisition commencing 50-min post-injection. PET scans were acquired on a Philips TF64 PET/CT. Low-dose CT was obtained for attenuation correction. All participants had a structural 3T MRI on a Siemens Skyra scanner to obtain high-resolution T1-weighted anatomical magnetization-prepared rapid gradient echo (MPRAGE) sequences.

### Image analysis

Centiloid values were computed from Aβ images using CapAIBL (https://milxcloud.csiro.au/tools/capaibl) [22]. All participants with Centiloid < 25 were classified as having a Aβ− PET result.

Tau PET scans were spatially normalized using the CapAIBL PCA-based approach [23]. Tau PET scans were scaled using the cerebellum cortex as the reference region. A gray matter inclusion mask and a meninges exclusion mask were applied. Standardized uptake value ratios (SUVR) were generated for the entorhinal cortex, amygdala, hippocampus, and parahippocampal gyrus, as well as a three composite ROI: mesial temporal (Me) (comprising the entorhinal cortex, amygdala, hippocampus, and parahippocampal gyrus), temporoparietal (Te) (comprising inferior and middle temporal, fusiform, supramarginal, and angular gyri, posterior cingulate/precuneus, superior and inferior parietal, and lateral occipital cortices), and rest of the neocortex (R) (comprising dorsolateral and ventrolateral prefrontal, orbitofrontal cortex, gyrus rectus, superior temporal, and anterior cingulate) [24]. Two thresholds were used to identify participants with higher vs lower MTL tau (i.e., higher vs lower Me SUVR). The cohort was ranked on their Me SUVR and the 90% percentile (90%ile) was used as a cut-off to separate the group into the top 10% and lower 90%, while the 95% percentile (95%ile) was used as a cut-off to separate the group into the top 5% and lower 95%. A third visually derived threshold, previously described [25], was also used to discriminate high (EC+) and low (EC−) tau tracer retention in the entorhinal cortex. The higher prevalence of tracer binding in the trans-entorhinal/entorhinal area compared to other regions elevated the 90th and 95th percentile thresholds such that they were not detecting visually apparent focal binding in the region. Consequently, a visual threshold was established for this region.

### Image sub-analysis

Six alternative reference regions were evaluated: cerebellar white matter, whole cerebellum, whole cerebellum plus pons, and three subcortical white matter reference regions. Evaluation was aimed at identifying the reference region with the lowest variance (standardized uptake value [SUV] standard deviation) and minimal outliers (measured by kurtosis) across the cohort, and the region for which there was no significant difference in SUV (independent samples *t*-test, *p* < 0.05) between the participants in the high MTL/entorhinal tau versus low MTL/entorhinal tau groups, as identified using all three thresholds.

### Neuropsychology assessment

All participants completed the full AIBL neuropsychology battery, as has been previously described [21]. The Mini-Mental State Examination (MMSE) and three cognitive composite scores were used as cognitive outcome measures to assess global cognition, memory, and non-memory domains of cognition, as well as early cognitive changes in AD. The composite memory score (CMS) comprised the participants’ scores on California Verbal Learning Test II (CVLT-II) long delay, Rey Complex Figure Test (RCFT) long delay, and the Logical Memory (LM) long delay. The composite non-memory score (CNMS) comprised the scores on RCFT copy, Boston Naming Test (30 items; BNT), Verbal Fluency (FAS total score), digit span total, digit symbol (coding), and category fluency (animals and boys names total score). An AIBL pre-clinical Alzheimer cognitive composite (AIBL-PACC) comprised the MMSE, coding, CVLT-II long delay, and LM long delay scores [26].

Raw scores were standardized using means and standard deviations of a group of 87 AIBL individuals who were cognitively unimpaired at their baseline visit and at 18-month follow-up (46% males, mean age 68.0 ± 3.7, mean education 15.1 ± 2.7, MMSE ≥ 28, CDR total and sum of boxes = 0, Geriatric Depression Score < 5) and were negative for Aβ, tau, and neurodegeneration (A-T-N-).

Participants were also classified as either memory complainers (subjective memory complaint, SMC) or memory non-complainers, based on their response to the question, “Do you have difficulties with your memory?” [21]

### Statistical analysis

All data were analyzed using SPSS version 27. Categorical data were analyzed using either chi-square tests of independence or Fisher’s exact test, where appropriate. Continuous data were analyzed using independent samples *t*-tests and Pearson’s correlation coefficients with a significance level of 0.05 (one-tailed, unless otherwise specified). Effect size is reported as Cohen’s *d*. Multiple linear regression was conducted in separate iterations, using the MMSE and each cognitive composite score (CMS, CNMS, AIBL-PACC) as the dependent variable, with age and Me SUVR as the independent variables. The false discovery rate approach was used to correct for multiple comparisons.

## Results

### Participants

**Fig. 1:**
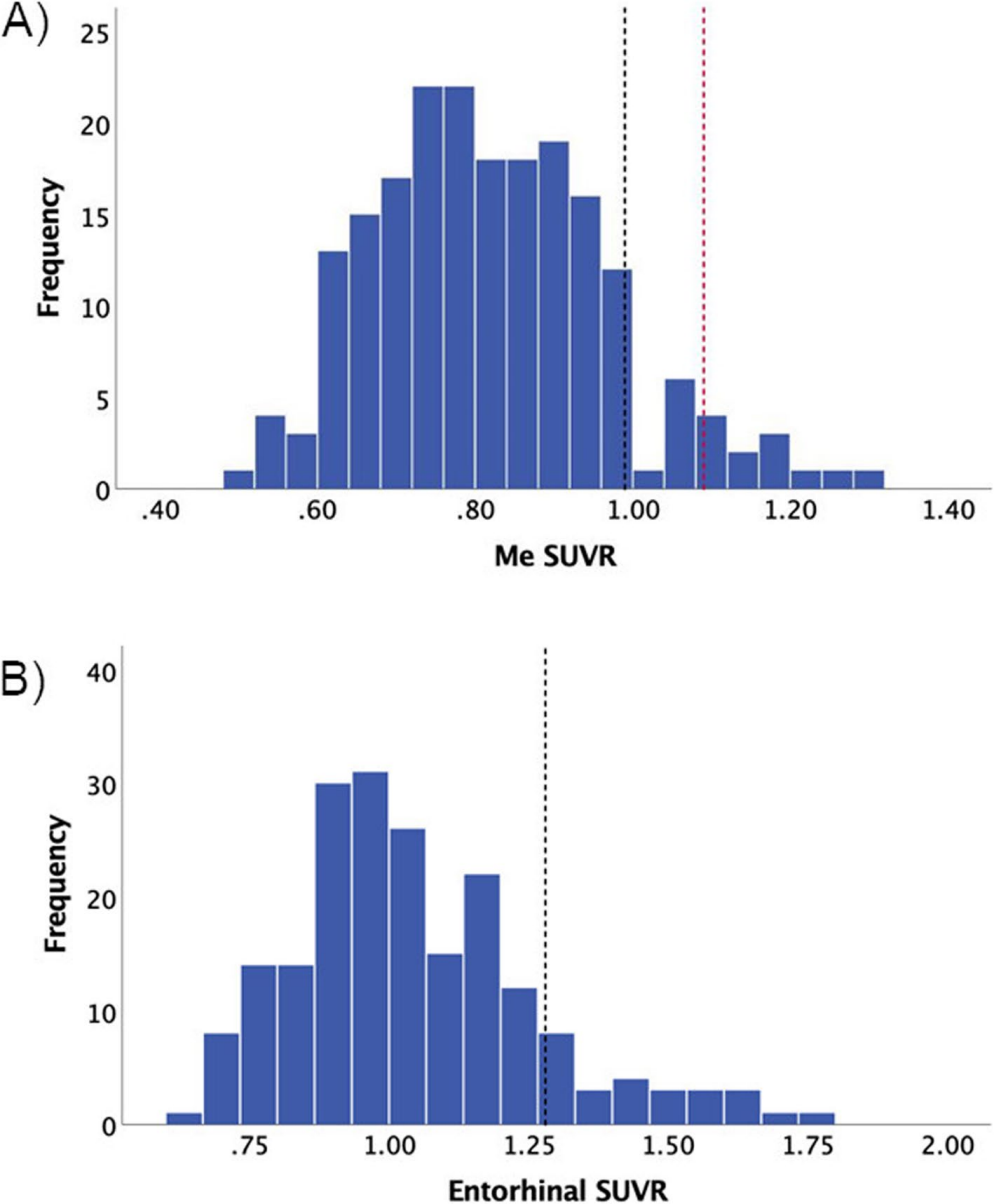
Histograms of mesial temporal and entorhinal cortex SUVR. Tau burden in a mesial temporal composite (Me) and the entorhinal cortex as measured by tau PET SUVR. **A** The red dashed line separates the cohort by the 95% percentile Me SUVR (top 5% vs lower 95%), while the black dashed line separates the cohort by the 90% percentile Me SUVR (top 10% vs lower 90%); **B** the black dashed line represents a visually derived threshold used to discriminate higher (EC+) from lower entorhinal SUVR (EC−)

One hundred and ninety-nine Aβ− CU participants were included in this study. Participants had a stable clinical classification for an average of 5.3 years (± 4.1) prior to and at the time of their tau PET scan. Figure 1 shows the distribution of Me SUVR and entorhinal SUVR across the entire cohort.

Demographics and characteristics of the cohort are shown in Table 1, split by the 90% percentile Me SUVR (lower 90% and top 10%). Participants with higher MTL tau were significantly older than participants with lower MTL tau. Participants with higher MTL tau also had lower hippocampal volumes, which remained significant after correction for age. The results were similar using the 95%ile threshold and the visually derived entorhinal cortex threshold (see Supplementary Tables 1 and 2, Additional files 1 and 2).

**Table 1:** Demographics and characteristics of the Aβ− cognitively unimpaired cohort split by the 90%ile Me SUVR

	90%ile Me SUVR	
	Lower 90% (***n = 179)***	Top 10% (***n = 20)***
**Age (years)**	74.3±5.0	78.3±5.7**
**Sex, F** ***n*** **(%)**	99 (55.3%)	14 (70.0%)
***APOE ε4*+,** ***n*** **(%)**^**a**^	40 (22.3%)	6 (30.0%)
**Education (years)**	14.3±3.1	13.5±3.2
**HV (cm**^**3**^**)**^**b**^	2.97±0.3	2.82±0.2**
**Centiloid**	2.02±7.0	3.36±11.1
**SMC,** ***n*** **(%)**	102 (57.0%)	11 (55.0%)

*Abbreviations*: *Me* mesial temporal composite, *SUVR* standardized uptake value ratio, *APOE* apolipoprotein E, *HV* hippocampal volume, *SMC* subjective memory complaint

Mean (SD), unless otherwise specified. *p ≤ 0.05, **p ≤ 0.01 compared to the lower 90% group

^a^*APOE* data was not available for 3 participants in the lower 90% group

^b^HV was only available for 17/20 participants in the top 10% group and 153/179 participants in the lower 90% group. Results remain significant after correction for age (*p* = 0.04); effect size, Cohen’s *d* = 0.34

**Fig. 2:**
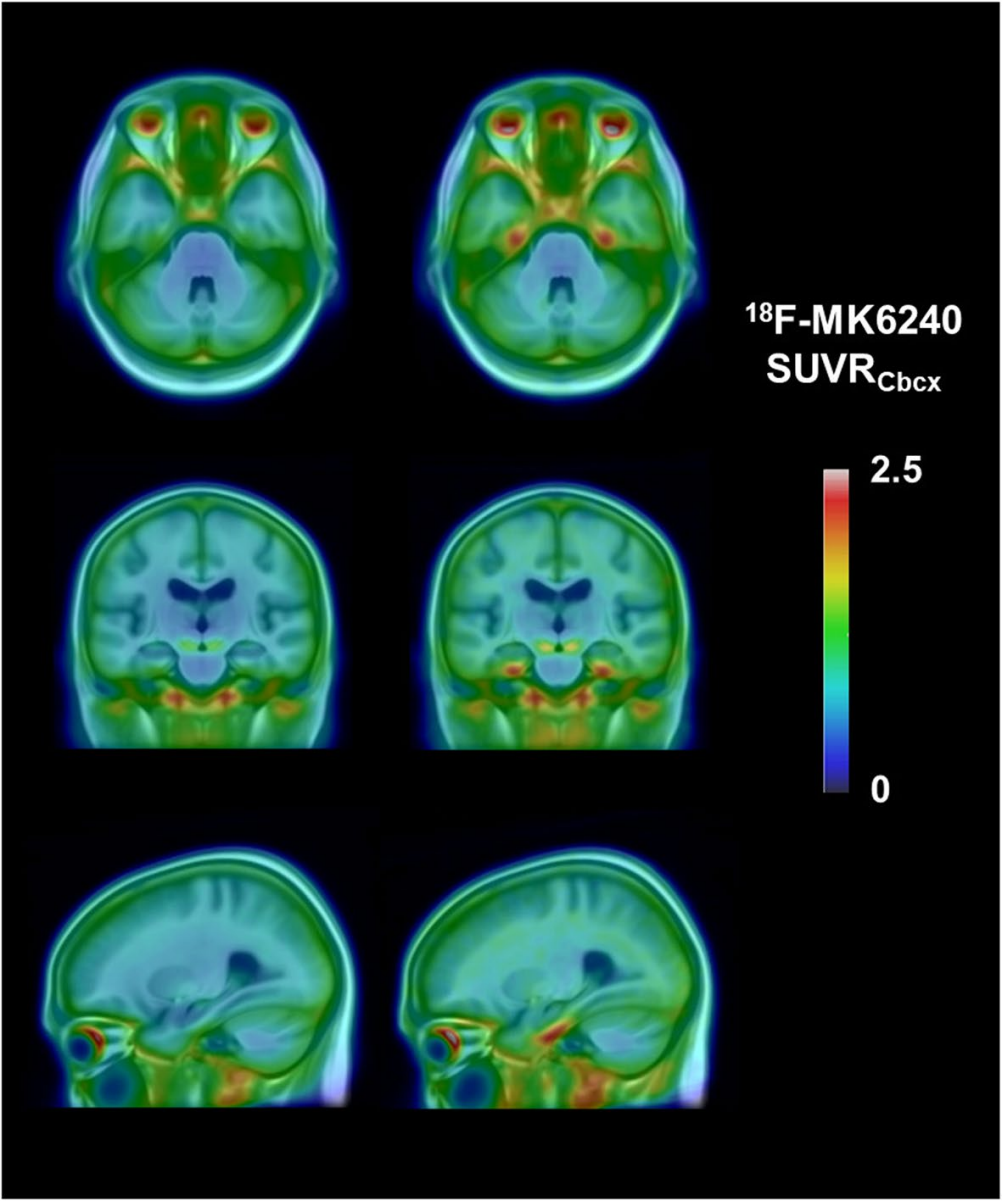
Mean tau ^18^F-MK6240 SUVR images for the cohort: lower 90% versus top 10%. Mean tau ^18^F-MK6240 SUVR images overlaid on a T1 MRI template for the cohort, lower 90% (left) and top 10% Me SUVR (right) showing tau tracer retention confined to Braak stages I–II

Using the thresholds specified, participants with higher MTL/higher entorhinal tau were visually observed to have a focal increase in tau tracer retention in a distribution consistent with Braak stages I–II when compared to participants with lower MTL/entorhinal tau (Fig. 2; Supplementary Figs. 1 and 2, Additional files 3 and 4). Additionally, these participants were visually observed to have a subtle increase in signal across the brain and extracerebral structures, though much less than the focal increase seen in the MTL.

Participants with high MTL tau also had a higher mean neocortical tau tracer retention (Te and R), which was significantly different compared to participants with lower MTL tau (Table 2). However, the mean images in Fig. 2 show that this increase is seen across the entire image including white matter and extracerebral structures suggesting a reference region problem.

**Table 2:** Mesial temporal tau and neocortical tau (cerebellar cortex reference region)

	90%ile Me SUVR			
	Lower 90% ***(n=179)***	Top 10% ***(n=20)***	***p***-value	Effect size
**Composite ROI (SUVRcbcx)**				
**Me SUVR**	0.79±0.11	1.12±0.08	***p*** **< 0.001**	*d = +2.97*
**Te SUVR**	0.99±0.12	1.14±0.09	***p*** **< 0.001**	*d = +1.24*
**R SUVR**	0.86±0.12	0.96±0.11	***p*** **< 0.001**	*d = +0.85*

Mean (SD). *t*-test (two-tailed). Effect size = Cohen’s *d. Abbreviations*: *Me* mesial temporal composite, *Te* temporoparietal composite, *R* rest of the neocortex composite

In a sub-analysis to determine whether differences in the reference region were driving these results, the groups were compared on their cerebellar cortex SUV. Participants in the top 10% Me SUVR were found to have significantly lower mean cerebellar cortex SUV values than participants in the lower 90% (*t* = 3.09, *p* = 0.001). Six alternative reference regions were evaluated. The alternative reference region with the lowest variance and for which the SUV did not differ between the top 10% and lower 90% groups (subcortical white matter) was selected, and composite ROI values were recalculated using this reference region, leaving the same participants classified as top 10% and lower 90%. The results showed that participants in the top 10% still had higher Me SUVR than the lower 90% (as expected), but participants in the top 10% no longer had higher neocortical (Te, R) SUVR values (Table 3). Table 3 shows that the lower 90% had significantly higher Te and R SUVR than the top 10%.

**Table 3:** Mesial temporal tau and neocortical tau (subcortical white matter reference region)

	90%ile Me SUVR			
	Lower 90% ***(n=179)***	Top 10% ***(n=20)***	***p***-value	Effect size
**Composite ROI (SUVRswm)**				
**Me**	1.11±0.13	1.30±0.23	***p*** **= 0.001**	*d= +1.36*
**Te**	1.41±0.18	1.32±0.16	***p*** **= 0.029**	*d= −0.52*
**R**	1.22±0.16	1.11±0.17	***p*** **= 0.005**	*d= −0.68*

Mean (SD). *t*-test (two-tailed). Effect size = Cohen’s *d. Abbreviations*: *Me* mesial temporal composite, *Te* temporoparietal composite, *R* rest of the neocortex composite

### Mesial temporal tau was associated with age

**Fig. 3:**
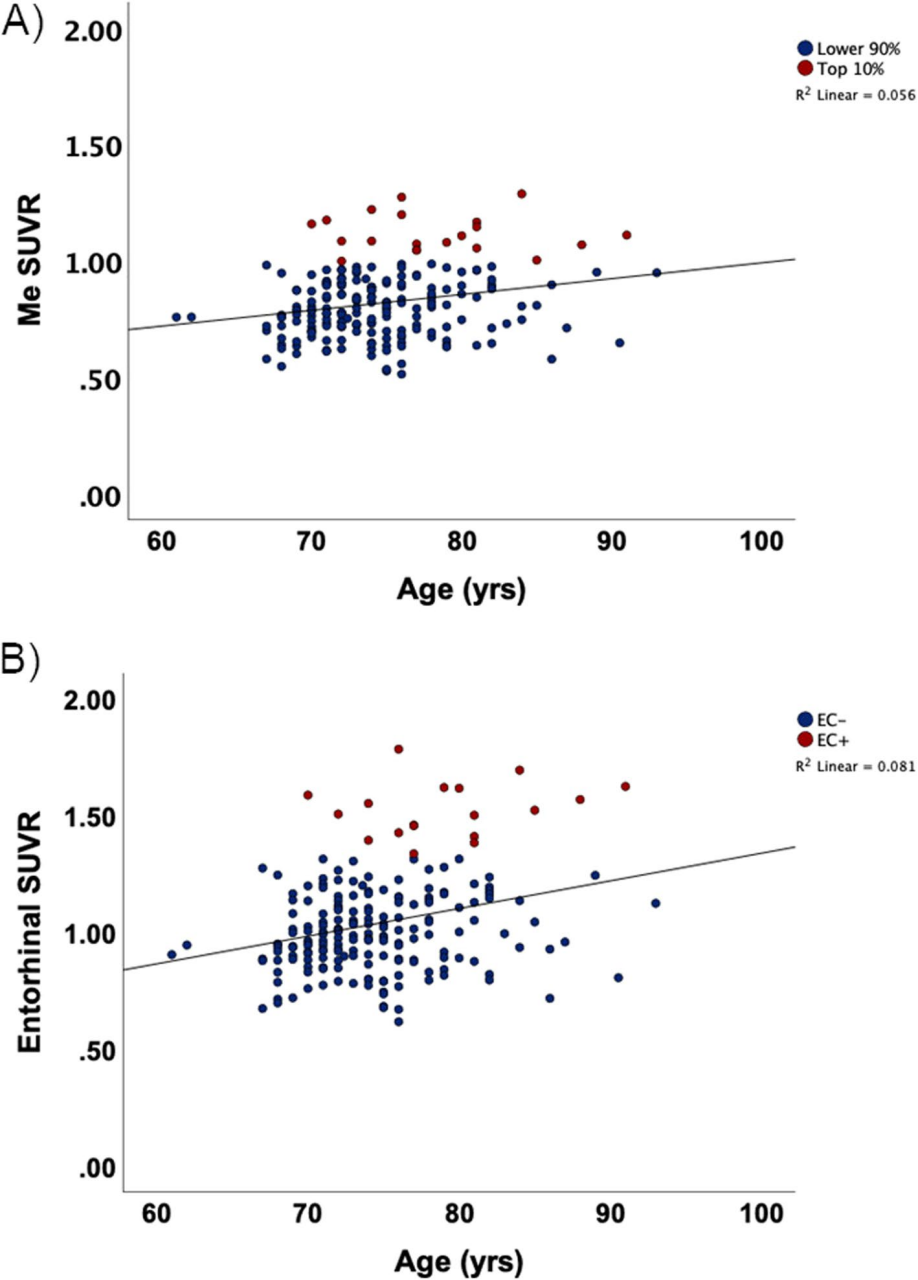
Scatterplots of mesial temporal SUVR and entorhinal SUVR versus age. Scatterplots showing **A** the correlation between age and mesial temporal (Me) SUVR and **B** the correlation between age and entorhinal SUVR

There was a significant association between age and Me SUVR (*r* = 0.24, *p* < 0.001) (Fig. 3A), but no association between age and neocortical SUVR (Te, *r* = −0.03, *p* = 0.33; R, *r* = −0.09, *p* = 0.10). For mesial temporal sub-regions, there was an association between age and SUVR generated for the entorhinal cortex (*r* = 0.29, *p* < 0.001) (Fig. 3B), amygdala (*r* = 0.21, *p* = 0.002), hippocampus (*r* = 0.21, *p* = 0.002), and parahippocampal gyrus (*r =* 0.14, *p* = 0.02).

### Mesial temporal tau was not associated with Aβ

There was no association between Aβ burden (measured in Centiloids) and Me SUVR (*r* = 0.08, *p* = 0.13). There was also no association between Centiloids and tau SUVR generated for Me sub-regions (entorhinal cortex, *r* = 0.05, *p* = 0.23; amygdala, *r* = 0.10, *p* = 0.07; hippocampus, *r* = 0.09, *p* = 0.11; and parahippocampal gyrus, *r* = 0.07, *p* = 0.17). Re-defining Aβ negative as less than 10 Centiloids did not affect the Me SUVR 90th or 95th percentile thresholds (see Supplementary Figs. 3 and 4, Additional file 5).

### Mesial temporal tau burden did not differ for individuals with and without subjective memory complaint

Across the cohort, 113/199 (56.8%) of participants had a subjective memory complaint (SMC). There was no significant difference in Me SUVR or entorhinal SUVR for individuals who had SMC compared to those who did not.

### Age but not tau burden is associated with worse cognition

Participants who were in the top 10% for Me SUVR and those who were EC+ did not have significantly different MMSE and composite memory scores compared to the other participants. EC+ also did not have significantly different AIBL-PACC scores than EC−. The top 10% had worse CNMS (*t* = 1.83, *p* = 0.03) and AIBL-PACC scores (*t* = 1.79, *p* = 0.04) than the lower 90%. Using the 95%ile threshold, the top 5% Me SUVR did not differ significantly than the lower 95% on any of the cognitive scores. However, as noted earlier, the higher MTL tau groups were significantly older than the lower MTL tau groups.

There was no correlation between cognitive performance (MMSE, CMS, CNMS, and AIBL-PACC) and SUVR in Me or the four Me sub-regions, both with and without the covariate of age.

Models combining age and Me SUVR were overall significant in accounting for the variance in MMSE, CNMS, and AIBL-PACC scores. However, these models accounted for only 4% of the variance in the MMSE (*R*^*2*^ = 0.04), 1.8% of the variance in CMS (*R*^*2*^ = 0.018), 10% of the variance in CNMS (*R*^*2*^ = 0.10), and 7.5% of the variance in the AIBL-PACC (*R*^*2*^ = 0.075). Of the variance explained, age was a significant contributor, while Me SUVR did not contribute significantly (Table 4). The results were similar with entorhinal cortex SUVR and age as independent variables and cognitive score as dependent variables (see Supplementary Table 3, Additional file 6).

**Table 4:** Multiple linear regression models of the relationship between Me SUVR, age, and cognitive composite scores

	***β***	***SE***	***t***	***p-value***
***Dependent variable = MMSE***				
**Age**	−0.20	0.02	−2.75	**0.007**
**Me SUVR**	−0.001	0.60	−0.008	0.99
***Dependent variable = CMS***				
**Age**	−0.12	0.01	−1.66	0.10
**Me SUVR**	−0.04	0.43	−0.49	0.62
***Dependent variable = CNMS***				
**Age**	−0.33	0.01	−4.69	**<0.001**
**Me SUVR**	0.03	0.30	0.38	0.71
***Dependent variable = AIBL-PACC***				
**Age**	−0.27	0.01	−3.77	**<0.001**
**Me SUVR**	−0.03	0.40	−0.40	0.69

*Abbreviations*: *β* standardized beta coefficient, *SE* standard error, *Me* mesial temporal, *SUVR* standardized uptake value ratio, *MMSE* Mini-Mental State Examination, *CMS* composite memory score, *CNMS* composite non-memory score, *PACC* pre-clinical Alzheimer cognitive composite

## Discussion

This cross-sectional study using the second-generation tau tracer ^18^F-MK6240 assessed the association between MTL tau and age, neocortical tau, and cognition in 199 Aβ− CU individuals. Visually, tau tracer retention was observed in a distribution consistent with, and limited to, Braak stages I–II. This observation was present whether Aβ negative was defined as less than 25 Centiloids or less than 10 Centiloids, and there was no correlation between Centiloid and Me SUVR values. This strongly suggests that the focal tau deposition observed in this study was independent of the Aβ plaque burden.

Participants with higher entorhinal/MTL tau appeared to have higher neocortical tau (Te, R) SUVR, as previously reported in studies of Aβ− CU with the tau tracer ^18^F-AV1451 (flortaucipir) [27]. However, evaluation of the reference region suggested that this observation was driven by subtle reductions in tau tracer retention in the cerebellar cortex in those with high MTL tau and no elevation was present in the neocortex when the subcortical white matter was used for normalization. Conversely, with the subcortical white matter reference region, while the MTL signal remained elevated, the neocortical areas were significantly and unexpectedly lower in the top 10% cohort. This highlights the difficulties of quantifying very slight changes in tau and the need for careful evaluation of the reference region when creating SUVR measures of regions of interest with relatively low tracer binding.

Entorhinal/MTL tau deposition was associated with age. While age contributed to some of the variance in cognitive scores, tau in these regions did not have an independent adverse impact on cognition. Additionally, while more than 50% of the cohort had a subjective memory complaint (SMC), these participants did not differ on entorhinal/MTL tau compared to individuals without a SMC. Results using the MTL tau thresholds were consistent with findings using the EC threshold, suggesting that MTL SUVR might be largely driven by the EC tau signal.

The association between entorhinal tau/MTL tau and age is consistent with findings from post-mortem evaluation [1–3, 13] and in vivo studies using tau PET [14, 27]. The lack of association between entorhinal/MTL tau and cognition is inconsistent with some prior tau PET studies [14, 15, 17, 27, 28], but unsurprising when we consider the cohort in this study and neuropathology reports. From post-mortem reports, cognitive impairment in Aβ− individuals was observed in association with more extensive tau burden (Braak stage ≥ III) [12, 13]. Here we report on a cohort of individuals who are both Aβ− and had a stable clinical classification of CU, for an average of 5.3 years (± 4.1) prior to their tau PET scan. This feature of this cohort, as well as differences in Aβ PET tracers used and the more limited tau distribution observed in this study than in prior studies, limits direct comparison to previous tau PET studies.

Subjective memory decline (SMD) has been identified as a risk factor for progression to dementia [29] and, in Aβ+ cognitively normal individuals, has been linked to a higher rate of progression to MCI or AD dementia [30]. In contrast to the findings in this study, SMD has previously been shown to be associated with higher entorhinal tau, after accounting for Aβ burden [31]. However, methods vary in the operationalization of SMC or SMD across studies. A single response question, as in this study, may not be as discriminatory in identifying those with subjective complaints compared to eliciting a report of decline in memory and administration of validated questionnaires to derive a composite [31].

In the absence of Aβ, tau in mesial temporal regions has been associated with atrophy in these regions [13, 14, 32]. In autopsy-confirmed cases of definite PART, tau NFT predominantly limited to Braak stages I–III has been associated with atrophy of the head of the left hippocampus [13], and medial temporal lobe atrophy has been significantly correlated with Braak stage, after correction for age [32]. In these cases, mesial temporal tau and atrophy were associated with cognitive impairment, with increasing Braak stage [13, 32]. Though there may be resilience factors at play in this cohort, the observation of mesial temporal tau and atrophy in this CU cohort suggests that both these processes may be occurring even before adverse effects on cognition are observed. Transactive response DNA-binding protein 43 kDa (TDP-43) co-occurs with tau NFT in the hippocampus with aging [33] and PART [13, 34]. TDP43 and tau NFT have been observed to have independent effects on hippocampal atrophy [35]. The effect of TDP43 on hippocampal atrophy cannot be evaluated in this study.

### Limitations

Our findings should be interpreted with caution, due to some limitations. By selecting CU participants for this study, we invariably restricted the variance in the cognitive scores. While this study had a reasonable sample size, the actual effect of MTL tau on cognition may have been too small to be detected in this study. On the one hand, increasing the sample size may improve the ability to detect this effect, if one exists; however, if the effect of MTL tau is small, then it is unlikely to be clinically meaningful. Participants in this study were motivated volunteers with high levels of education, few medical comorbidities, and familiar with the cognitive assessments administered (owing to the serial evaluations they undertook); therefore, these results may not be broadly generalizable. Additionally, this study is limited by the lack of a replication cohort to validate these findings.

## Conclusions

In this clinically stable Aβ− CU cohort, tau tracer retention was consistent with the distribution of Braak stages I–II and age-related, but not associated with sub-threshold Aβ levels. Tau deposition in these regions was associated with smaller hippocampal volumes, but did not have an adverse effect on cognition, after accounting for age.

## Supplementary Information

**Additional file 1: Supplementary Table 1.** Demographics and characteristics of the cohort split by the 95%ile Me SUVR. Description of data - Demographics and characteristics of the cohort split by the 95%ile Me SUVR

**Additional file 2: Supplementary Table 2.** Demographics and characteristics of the cohort split by the visually derived entorhinal cortex threshold. Description of data - Demographics and characteristics of the cohort split by the visually derived entorhinal cortex threshold

**Additional file 3: Supplementary Figure 1**. Mean tau 18F-MK6240 SUVR images for the cohort: lower 95% versus top 5%. Description of data - Mean tau 18F-MK6240 SUVR images for the cohort: lower 95% versus top 5%

**Additional file 4: Supplementary Figure 2.** Mean tau 18F-MK6240 SUVR images for the cohort: visually derived EC- versus EC+. Description of data - Mean tau 18F-MK6240 SUVR images for the cohort: visually derived EC- versus EC+

**Additional file 5: Supplementary Figures 3 and 4.** Mean tau 18F-MK6240 SUVR images for participants with Centiloid less than 10 – lower 90% versus top 10% Me SUVR (Supplementary figure 3); and mean tau 18F-MK6240 SUVR images for participants with Centiloid less than 10 – lower 95% versus top 5% Me SUVR (Supplementary figure 4).

**Additional file 6: Supplementary Table 3.** Multiple linear regression models of the relationship between entorhinal SUVR, age and cognitive composite scores. Description of data - Multiple linear regression models of the relationship between entorhinal SUVR, age and cognitive composite scores in the table and additional paragraph describing the data

## Data Availability

The datasets used and/or analyzed during the current study are available from the corresponding author upon reasonable request.

## Abbreviations

MTL: Mesial temporal lobe
Aβ: Amyloid-β
HV: Hippocampal volume
CU: Cognitively unimpaired
SUVR: Standardized uptake value ratio
Me: Mesial temporal composite
Te: Temporoparietal composite
R: Rest of the neocortex composite
NFT: Neurofibrillary tangles
EC: Entorhinal cortex
AD: Alzheimer’s disease
PART: Primary age-related tauopathy
SUV: Standardized uptake value
MMSE: Mini-Mental State Examination
CMS: Composite memory score
CNMS: Composite non-memory score
AIBL-PACC: Australian Imaging Biomarkers and Lifestyle Study of Ageing pre-clinical Alzheimer cognitive composite
SMC: Subjective memory complaint
*APOE*: Apolipoprotein E
SMD: Subjective memory decline
TDP-43: TAR DNA-binding protein 43
