# Supplementary material for: Mesial temporal tau in amyloid-β-negative cognitively normal older persons
Source: Alzheimers Res Ther. 2022 Apr 8;14:51. doi: 10.1186/s13195-022-00993-x (PMC8991917; doi:10.1186/s13195-022-00993-x)
Supplement: Supplementary file 1 — Additional file 1: Supplementary Table 1. Demographics and characteristics of the cohort split by the 95%ile Me SUVR. Description of data - Demographics and characteristics of the cohort split by the 95%ile Me SUVR [file 13195_2022_993_MOESM1_ESM.docx]

**Supplementary Table 1 Demographics and characteristics of the cohort split by the 95%ile Me SUVR**

|  | **95%ile Me SUVR** | |
| --- | --- | --- |
|  | **Lower 95%**  ***(n = 189)*** | **Top 5%**  ***(n =10)*** |
| **Age (y)** | 74.5±5.1 | 78.4±6.3* |
| **Sex, F *n* (%)** | 107 (56.6%) | 6 (60%) |
| ***APOE ε4*+, *n* (%)^a^** | 41 (21.7%) | 5 (50%) |
| **Education (y)** | 14.3±3.1 | 14.0±3.5 |
| **HV (cm^3^)^b^** | 2.97±0.3 | 2.75±0.2** |
| **Centiloid** | 1.89±7.2 | 7.12±11.1 |
| **SMC, *n* (%)** | 106 (56.1%) | 7 (70%) |

Abbreviations: Me = mesial temporal composite; SUVR = standardized uptake value ratio; *APOE* = Apolipoprotein E; HV = hippocampal volume; SMC = subjective memory complaint.

Mean (SD), unless otherwise specified. *p≤0.05, **p≤0.01 compared to the lower 95%.

^a^ *APOE* data was not available for 3 lower 95% participants.

^b^ HV was only available for 160/189 lower 95% and 9/10 top 5% participants. Results remain significant after correction for age (p=0.02); effect size Cohen’s d = 0.72.
