# Supplementary material for: Mesial temporal tau in amyloid-β-negative cognitively normal older persons
Source: Alzheimers Res Ther. 2022 Apr 8;14:51. doi: 10.1186/s13195-022-00993-x (PMC8991917; doi:10.1186/s13195-022-00993-x)
Supplement: Supplementary file 2 — Additional file 2: Supplementary Table 2. Demographics and characteristics of the cohort split by the visually derived entorhinal cortex threshold. Description of data - Demographics and characteristics of the cohort split by the visually derived entorhinal cortex threshold [file 13195_2022_993_MOESM2_ESM.docx]

**Supplementary Table 2 Demographics and characteristics of the cohort split by the visually derived entorhinal cortex threshold**

| **Threshold** | **Visually derived EC threshold** | |
| --- | --- | --- |
|  | **EC-**  ***(n=181)*** | **EC+**  ***(n=18)*** |
| **Age (y)** | 74.3±5.0 | 79.1±5.5** |
| **Sex, F *n* (%)** | 100 (55.2%) | 13 (72.2%) |
| ***APOE ε4*+, *n* (%)^a^** | 42 (23.2%) | 4 (22.2%) |
| **Education (y)** | 14.3±3.1 | 13.4±3.2 |
| **HV (cm^3^)^b^** | 2.97±0.3 | 2.82±0.2* |
| **Centiloid** | 2.07±7.2 | 3.03±10.3 |
| **SMC, *n* (%)** | 102 (56.4%) | 11 (61.1%) |

Abbreviations: Me = mesial temporal composite; SUVR = standardized uptake value ratio; *APOE* = Apolipoprotein E; HV = hippocampal volume; SMC = subjective memory complaint.

Mean (SD), unless otherwise specified. *p≤0.05, **p≤0.01 compared to EC-.

^a^*APOE* data was not available for 3 EC- participants.

^b^ HV was only available for 155/181 EC- and 15/18 EC+ participants. Results not significant after correction for age (p=0.19).
