# Supplementary material for: Mesial temporal tau in amyloid-β-negative cognitively normal older persons
Source: Alzheimers Res Ther. 2022 Apr 8;14:51. doi: 10.1186/s13195-022-00993-x (PMC8991917; doi:10.1186/s13195-022-00993-x)
Supplement: Supplementary file 3 — Additional file 3: Supplementary Figure 1. Mean tau 18F-MK6240 SUVR images for the cohort: lower 95% versus top 5%. Description of data - Mean tau 18F-MK6240 SUVR images for the cohort: lower 95% versus top 5% [file 13195_2022_993_MOESM3_ESM.docx]

**Supplementary Figure 1. Mean tau ^18^F-MK6240 SUVR images for the cohort: lower 95% versus top 5%**

**
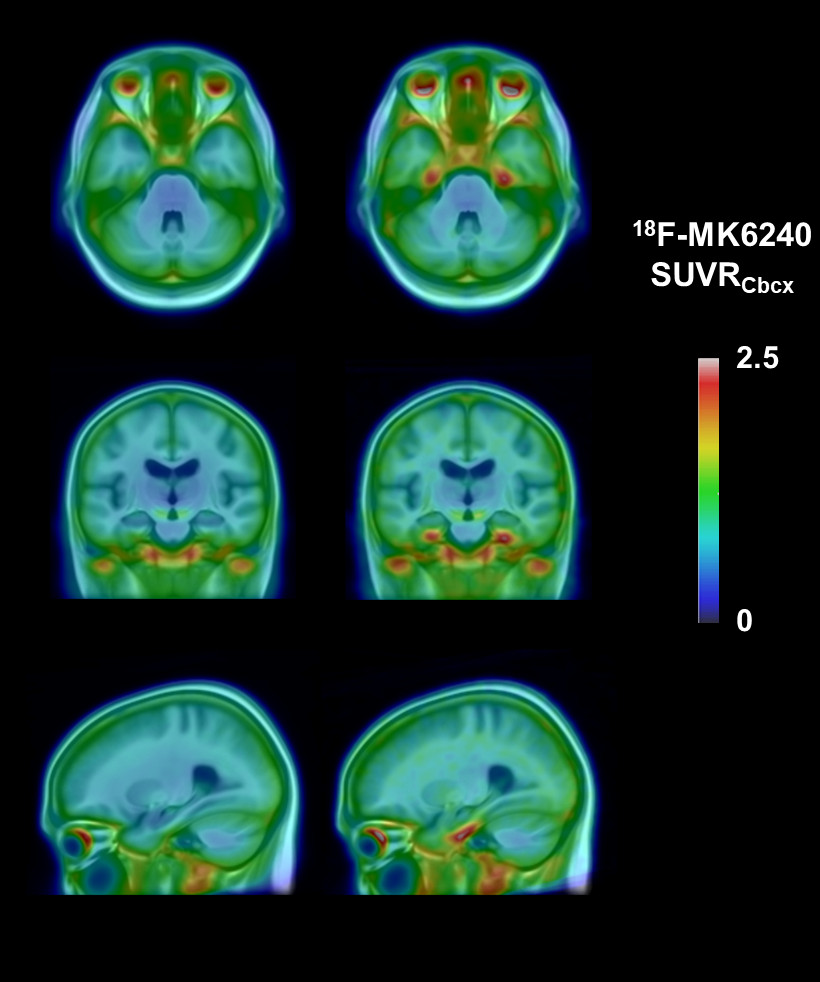
**

Mean tau ^18^F-MK6240 SUVR images overlaid on a T1 MRI template for the cohort, lower 95% (left) and top 5% Me SUVR (right) showing tau tracer retention confined to Braak stage I-II.
