# Supplementary material for: Mesial temporal tau in amyloid-β-negative cognitively normal older persons
Source: Alzheimers Res Ther. 2022 Apr 8;14:51. doi: 10.1186/s13195-022-00993-x (PMC8991917; doi:10.1186/s13195-022-00993-x)
Supplement: Supplementary file 4 — Additional file 4: Supplementary Figure 2. Mean tau 18F-MK6240 SUVR images for the cohort: visually derived EC- versus EC+. Description of data - Mean tau 18F-MK6240 SUVR images for the cohort: visually derived EC- versus EC+ [file 13195_2022_993_MOESM4_ESM.docx]

**Supplementary Figure 2. Mean tau ^18^F-MK6240 SUVR images for the cohort: visually derived EC- versus EC+**

**
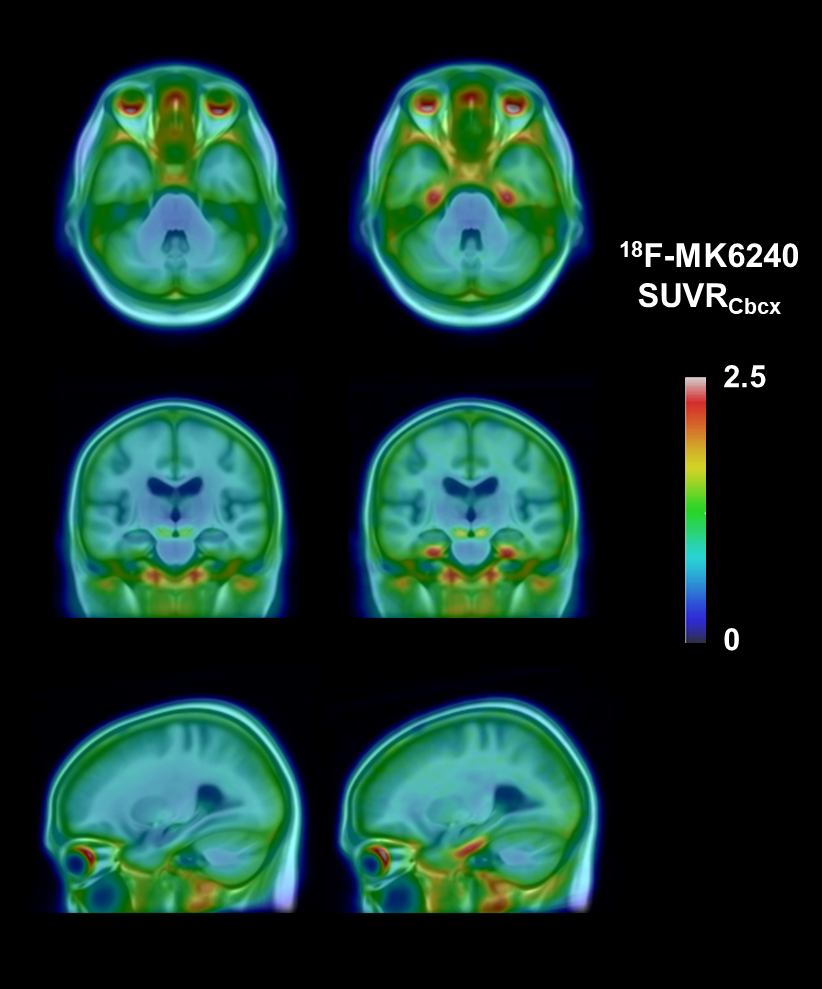
**

Mean tau ^18^F-MK6240 SUVR images overlaid on a T1 MRI template for the cohort, EC- (left) and EC+ (right) showing tau tracer retention confined to Braak stage I-II.

**2.5**

**0**
