# Supplementary material for: Mesial temporal tau in amyloid-β-negative cognitively normal older persons
Source: Alzheimers Res Ther. 2022 Apr 8;14:51. doi: 10.1186/s13195-022-00993-x (PMC8991917; doi:10.1186/s13195-022-00993-x)
Supplement: Supplementary file 5 — Additional file 5: Supplementary Figures 3 and 4. Mean tau 18F-MK6240 SUVR images for participants with Centiloid less than 10 – lower 90% versus top 10% Me SUVR (Supplementary figure 3); and mean tau 18F-MK6240 SUVR images for participants with Centiloid less than 10 – lower 95% versus top 5% Me SUVR (Supplementary figure 4). [file 13195_2022_993_MOESM5_ESM.docx]

**Supplementary Figure 3. Mean tau ^18^F-MK6240 SUVR images for the participants with Centiloid <10: lower 90% versus top 10%**


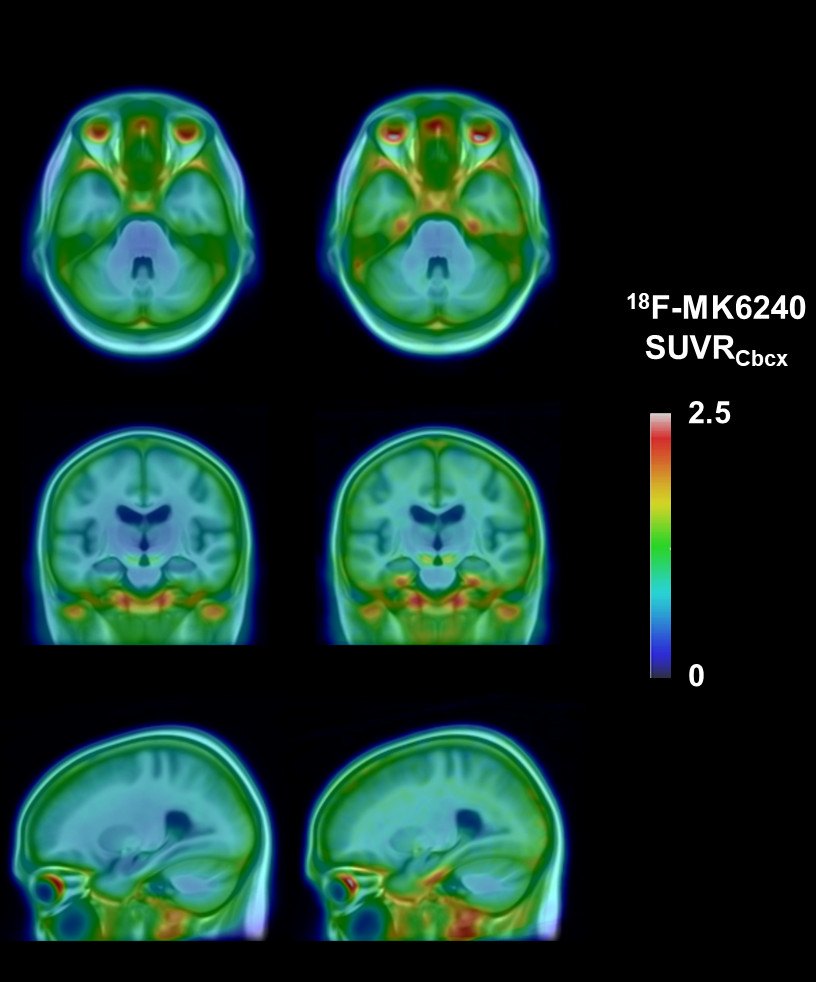


Mean tau ^18^F-MK6240 SUVR images overlaid on a T1 MRI template for participants with Centiloid <10, lower 90% (left) (n=157) and top 10% Me SUVR (right) (n=18) showing tau tracer retention confined to Braak stage I-II.

**Supplementary Figure 4. Mean tau ^18^F-MK6240 SUVR images for the participants with Centiloid <10: lower 95% versus top 5%**

**
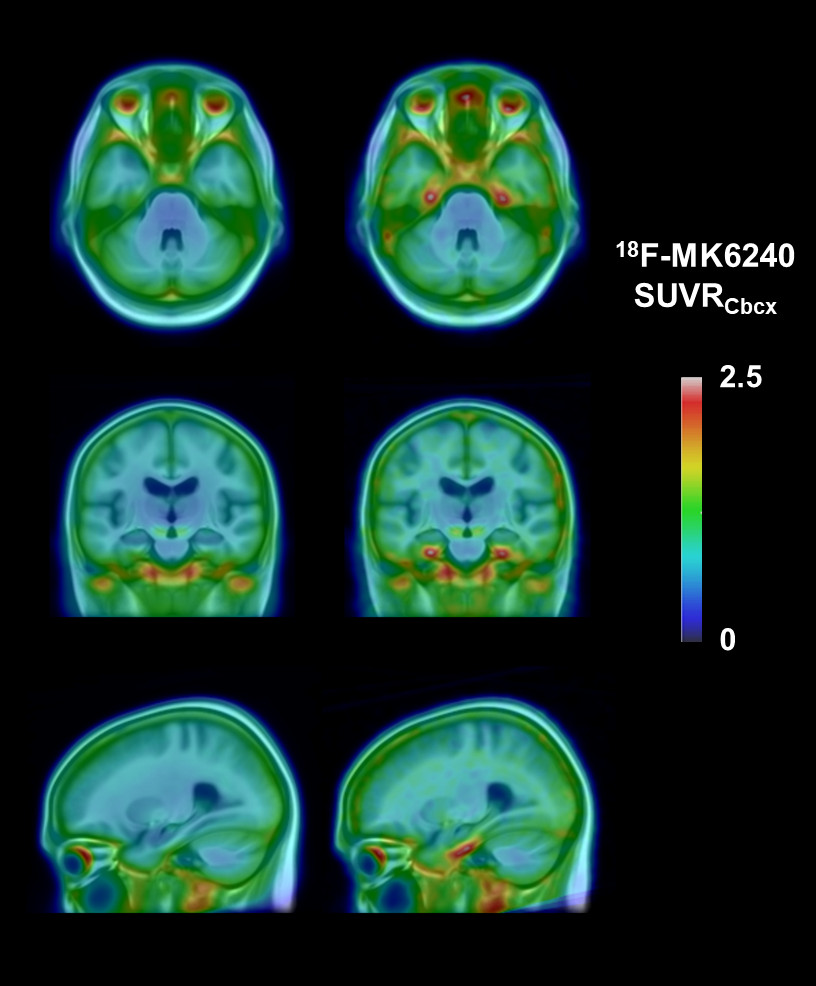
**

Mean tau ^18^F-MK6240 SUVR images overlaid on a T1 MRI template for participants with Centiloid <10, lower 95% (left) (n=166) and top 5% Me SUVR (right) (n=9) showing tau tracer retention confined to Braak stage I-II.

**2.5**

**0**
