# Supplementary material for: Mesial temporal tau in amyloid-β-negative cognitively normal older persons
Source: Alzheimers Res Ther. 2022 Apr 8;14:51. doi: 10.1186/s13195-022-00993-x (PMC8991917; doi:10.1186/s13195-022-00993-x)
Supplement: Supplementary file 6 — Additional file 6: Supplementary Table 3. Multiple linear regression models of the relationship between entorhinal SUVR, age and cognitive composite scores. Description of data - Multiple linear regression models of the relationship between entorhinal SUVR, age and cognitive composite scores in the table and additional paragraph describing the data [file 13195_2022_993_MOESM6_ESM.docx]

Models combining age and entorhinal SUVR were overall significant in accounting for the variance in MMSE, CNMS and the AIBL-PACC score. However, these models accounted for only 4% of the variance in the MMSE (*R^2^* = 0.04), 2% of the variance in CMS (*R^2^* = 0.02), 10% of the variance in CNMS (*R^2^* = 0.10) and 7.6% of the variance in the AIBL-PACC (*R^2^* = 0.076). Of the variance explained, age was a significant contributor, while Me SUVR did not contribute significantly (Supplementary Table 3).

**Supplementary Table 3. Multiple linear regression models of the relationship between entorhinal SUVR, age and cognitive composite scores**

|  |  | ***Dependent variable = MMSE*** | | |
| --- | --- | --- | --- | --- |
|  | ***β*** | ***SE*** | ***t*** | ***P value*** |
| **Age** | -0.20 | 0.02 | -2.73 | **0.007** |
| **EC SUVR** | 0.005 | 0.42 | 0.07 | 0.95 |
|  |  | ***Dependent variable = CMS*** | | |
| **Age** | -0.11 | 0.01 | -1.54 | 0.13 |
| **EC SUVR** | -0.06 | 0.30 | -0.77 | 0.45 |
|  |  | ***Dependent variable = CNMS*** | | |
| **Age** | -0.32 | 0.01 | -4.57 | **<0.001** |
| **EC SUVR** | 0.008 | 0.21 | 0.12 | 0.91 |
|  |  | ***Dependent variable = AIBL-PACC*** | | |
| **Age** | -0.26 | 0.01 | -3.68 | **<0.001** |
| **EC SUVR** | -0.03 | 0.28 | -0.47 | 0.64 |

Abbreviations: β = standardized beta coefficient; SE = standard error; EC = entorhinal cortex; SUVR = standardized uptake value ratio; MMSE = mini-mental state examination; CMS = composite memory score; CNMS = composite non-memory score; PACC = pre-clinical Alzheimer cognitive composite.
